# A Case in Practice

**Published:** 1876-01

**Authors:** J. M. Whitney


### A CASE IN PRACTICE.

BY J. M. WHITNEY.

*Read before the Ohio State Dental Society, Dec. 2d, 1875.*

About a year ago, a young man was under the care of one of the leading dental practitioners of Philadelphia, he had some, not very large, operations performed in the first and second molars of the left side.

Soon after the operation he felt a soreness with some appreciable signs of inflammation upon that side of the face. He returned to his dentist, who gave as his opinion, that the trouble arose from the gums about the lower *dens sapientia*, which was not yet irrupted, but around which there was much inflammation and swelling.

It was thoroughly lanced but only swelled with much more rapidity than before, and in a day or two he was confined to his bed.

One of the most skillful surgeons in the city, a professor in one of the medical colleges was called, but nothing that his skill could devise seemed in the least to check the extensive inflammation.

The swelling increased until, in a short time, it filled the entire space from the top of the head to the middle of the shoulder. His eyes were closed, nothing but liquids could be received into the mouth and thus he lay, for over two months.

Meantime the surgeon had made long and deep incisions, to prevent the pus, which formed in great quantities, from burrowing beneath the clavicle.

By the first of July, the inflammation and swelling had so far subsided that the surgeon gave his consent to his taking a trip to the West, making it by short and easy stages.

Before he reached Buffalo, the old pain returned, quickly followed by rapidly spreading inflammation and swelling, but being determined to reach my home, he made only a short stay and took the cars for Cleveland. On the cars the abscess broke, pouring out great quantities of pus, and he reached my house, pale and exhausted.

After a rest of some time, he, as well as his mother and aunt, who accompanied him, requested me to examine his case and give them my opinion.

I complied with their wishes and after a long and careful examination told them I thought the whole difficulty arose from the buried wisdom tooth, whose position in the jaw was similar to these specimens, lying almost horizontal, the crown pressing firmly against the second molar.

I advised extraction, to which he consented.

After sustaining him with stimulants, I dissected back mucous membrane and underlying tissue, cut away with chisel and sharp pointed alveolar forceps a portion of the alveolar process, threw the tooth upward a little with the physic forceps, raised it with a sharp pointed forcep and lifted it from its position. I found, as I expected, the roots shortened and their apices very rough.

He quickly ralied from the operation and by night said he had not felt so well since his illness began.

A letter received from his aunt two weeks ago, said that he had continued to improve from the date of the operation to the present time, with not the least return of his former pain, and had recently started for Europe.

My explanation as to why that tooth should have been the cause of such extensive inflammation and serious results is,

First, the young man was much exhausted from a long and severe course of study, so that his system was in the condition most favorably disposed to take on inflammatory action from any source of irritation.

Second, the *dens sapientia*, on account of its peculiar position, pressed with great force against the second molar. This produced an irritation not only in the mucous membrane, but at the end of the roots, where its persistance caused a breaking down of the tissue and formation of pus.

The roots being encased in thick strong walls, the pus was forced to remain about the roots for some time, before it could make for itself a passage to the softer tissue.

Meantime the roots were being roughened, so that they ever after continued to be sources of irritation, and as a natural consequence, fountains from which the pus would flow through the long sinuses that found the surface at different points, some just below the maxillary and others near the clavicle.

I think the near proximity to the inferior dental canal, may have had an influence in keeping up the irritation.

